# Surface Transfer Doping in MoO_3–*x*_/Hydrogenated Diamond Heterostructure

**DOI:** 10.1021/acs.jpclett.3c03541

**Published:** 2024-02-01

**Authors:** Liqiu Yang, Ken-ichi Nomura, Aravind Krishnamoorthy, Thomas Linker, Rajiv K. Kalia, Aiichiro Nakano, Priya Vashishta

**Affiliations:** † Collaboratory for Advanced Computing and Simulation, University of Southern California, Los Angeles, California 90089, United States; ‡ Department of Mechanical Engineering, Texas A&M University, College Station, Texas 77843, United States; § Stanford PULSE Institute, SLAC National Accelerator Laboratory, Menlo Park, California 94025, United States

## Abstract

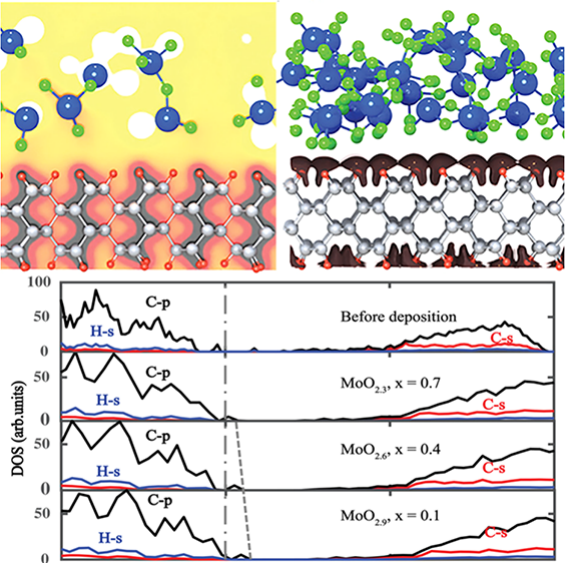

Surface transfer doping is proposed to be a potential solution for doping diamond, which is hard to dope for applications in high-power electronics. While MoO_3_ is found to be an effective surface electron acceptor for hydrogen-terminated diamond with a negative electron affinity, the effects of commonly existing oxygen vacancies remain elusive. We have performed reactive molecular dynamics simulations to study the deposition of MoO_3–*x*_ on a hydrogenated diamond (111) surface and used first-principles calculations based on density functional theory to investigate the electronic structures and charge transfer mechanisms. We find that MoO_3–*x*_ is an effective surface electron acceptor and the spatial extent of doped holes in hydrogenated diamond is extended, promoting excellent transport properties. Charge transfer is found to monotonically decrease with the level of oxygen vacancy, providing guidance for engineering of the surface transfer doping process.

With a wide bandgap of 5.5 eV, high carrier mobility (4,500 cm^2^/V s for electrons and 3,800 cm^2^/V s for holes),^1^ large breakdown electric field (exceeding 10 MV/cm), high thermal conductivity (22 W cm^–1^ K^–1^), and resistance to extreme environmental conditions, diamond-based field-effect transistors (FETs) are promising candidates for high-frequency and high-power electronics.^2^ Rising attention has been given to control the conductivity and transform the well-known insulator, diamond.^3^ However, compared with other semiconductors, it is hard to achieve traditional substitutional doping in diamond. Though p-type doping by boron and n-type doping by phosphorus or nitrogen are reported, the charge carrier densities are still low at room temperature.^4,5^ Surface transfer doping (STD) has been proposed as a potential solution, where doping is achieved by electron exchange at the interface between the diamond surface and the dopant solid.^3,6,7^ MoO_3_ and V_2_O_5_ are reported to be two of the most promising oxide materials for this STD process, potentially increasing their application for FETs.^8^

Diamond surfaces are reported to show negative electron affinity after hydrogen termination, which facilitates the electron transfer from diamond to the surface electron acceptors and creates a quasi-two-dimensional subsurface hole gas (2DHG).^9,10^ Hydrogenation of diamond can be achieved by several methods, including plasma-enhanced chemical vapor deposition (PECVD) and thermal hydrogenation.^11,12^ After hydrogenation, exposing diamond with surface dopants is straightforward, and the effect of STD will not disappear because of low bulk conductivity.^6^ MoO_3_ is an effective electron-accepting/hole-injection material, which improves the performance and stability of STD.^8^ While the hydrogenated diamond surface deposited with MoO_3_ was shown to exhibit high p-type surface conductivity, atomistic and electronic structures of the interface remain elusive. There is also a lack of knowledge on the effects of oxygen vacancies, which are commonly observed in MoO_3_ when exposed to atmosphere.^13^

Here, we perform first-principles-informed reactive molecular dynamics (RMD) simulations^14^ using ReaxFF interatomic potential to study the interfacial structure of the diamond–MoO_3–*x*_ interface. ReaxFF is designed to describe material properties as well as chemical reactions at density functional theory (DFT)-level accuracy based on the bond-order concept and charge equilibrium (QEq) scheme. ReaxFF has been used to simulate diamond,^15^ metal oxides,^16−25^ and organic–inorganic interfaces.^26−28^ Our ReaxFF simulations are performed to elucidate the deposition mechanism of MoO_3–*x*_ on a hydrogenated diamond (111) surface. Electronic density-of-states alignment and charge transfer at the interface are studied using first-principles calculations based on DFT for selected thermalized structures taken from the RMD simulation trajectories. We observe shifting of density-of-states alignment and higher charge transfer for higher Mo oxidation state. Such atomistic and electronic details provide mechanistic understanding of the surface transfer doping process.

*Interfacial Structure*. In Figure 1, we show side views of the structures of three systems. MoO_3_ has two thermodynamic phases, one being stable and the other being metastable. In this study, we use molten oxides for the deposition process. We also note that the MoO_3–*x*_/H-diamond interface changes with varying oxygen vacancy level, *x* = 0.1, 0.4, 0.7.

**Figure 1:**
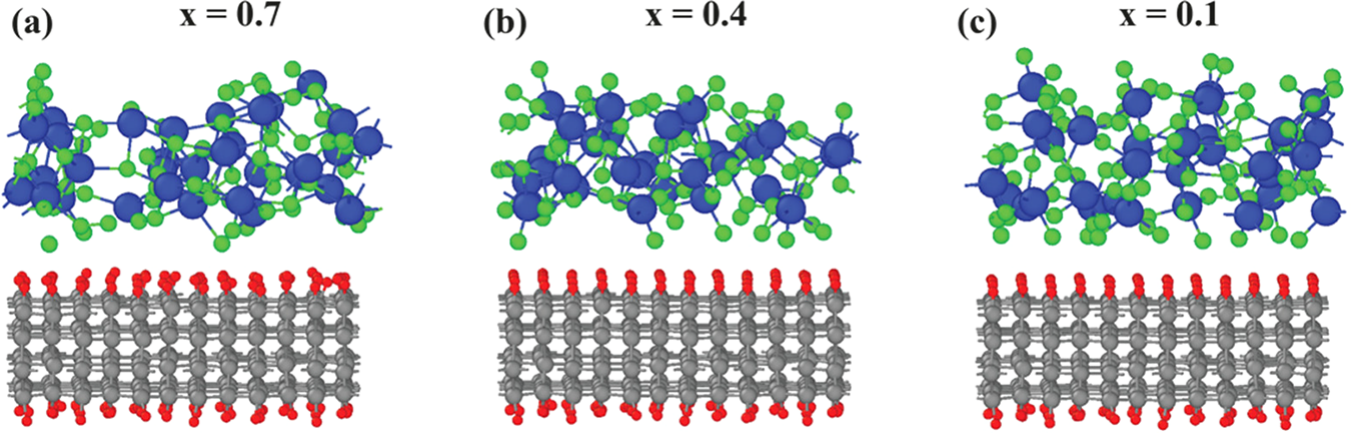
MoO_3–*x*_ on encapsulated H-diamond systems with value of *x* being 0.7 (a), 0.4 (b), and 0.1 (c). The gray, red, blue, and green spheres represent C, H, Mo, and O atoms, respectively.

We computed the Mo–O partial pair distribution for the three systems. Overall, pair distributions exhibit the highly disordered nature of the MoO_3–*x*_ structures. The Mo–O partial pair distribution shifts leftward as the vacancy level, *x*, increases, which suggests that as more vacancies are introduced into the material, the atoms that remain tend to get closer to each other on average. Atomic arrangement becomes more compact or denser as vacancy levels increase. This could be due to the atoms rearranging themselves to minimize the overall energy of the system in response to the introduction of oxygen vacancies. In addition, the Mo–O bond length distributions for the three systems are also quite informative. Along with the atomic arrangement getting more compact, the Mo–O bond length gets shorter with the increase of the oxygen vacancy level, indicating that the Mo–O bonds become stronger as the vacancy level increases. This is understandable since there is a smaller number of neighbor atoms for each atom for larger *x*, and accordingly, bonding strength is shared by a smaller number of bonds per atom to make each stronger.

*Charge Transfer*. To investigate the charge transfer process quantitatively, we first computed the Bader charges^29,30^ of atoms to calculate the charge difference after deposition. Figure 2 shows the Bader charges before and after depositing oxides on top of the hydrogenated diamond (111) surface. Upon deposition, the net computed electron charge derived from Table 1 for hydrogenated diamond is 1.6, 1.8, and 2.0 for *x* = 0.7, 0.4, and 0.1, respectively, with MoO_3–*x*_ having equal and opposite charge, demonstrating MoO_3–*x*_’s ability as an electron acceptor for different levels of oxygen vacancy. This illustrates a higher charge transfer for higher Mo oxidation state. Before deposition we consistently find that hydrogen is more electronegative (having negative Bader charge), which has been attributed to changing from loosely bound π bonding to tightly bound C–H σ bonding upon hydrogenation of diamond.^31^ Furthermore, the Bader analysis indicates the hole transfer is primarily localized to hydrogen, with the hydrogen atoms becoming more positively charged compared to before deposition and the carbon atoms becoming more negatively charged. As oxygen is strongly electronegative, the weak attractive interaction between oxygen and hydrogen at the interface drives this charge transfer.

**Figure 2:**
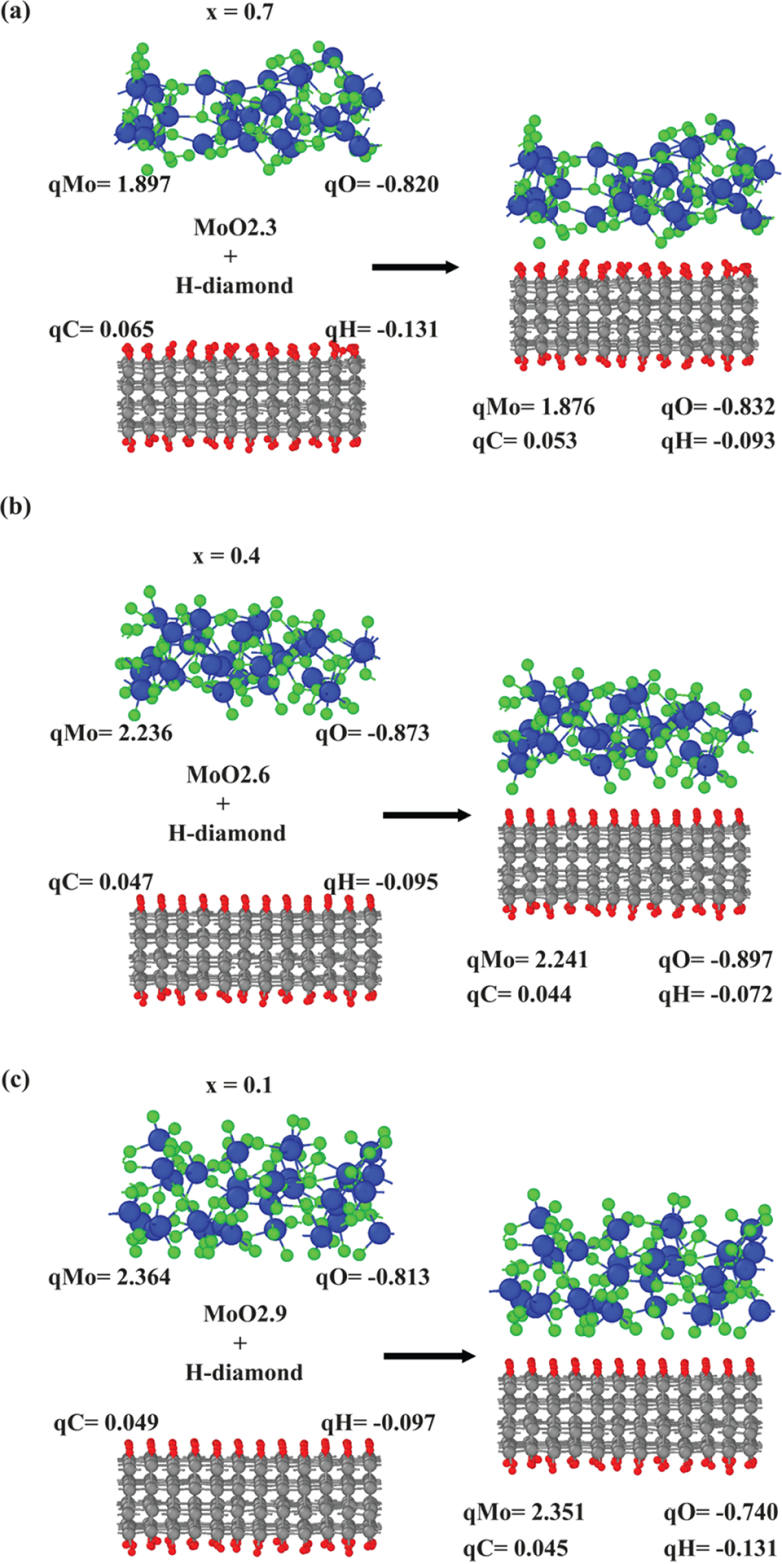
Average Bader charges of the atoms before and after deposition. Diamond-MoO_3–*x*_ systems with *x* being 0.7 (a), 0.4 (b), and 0.1 (c). The color scheme for the atoms is the same as that in Figure 1.

**Table 1:** Charge Transfer Per Atom Due to Deposition

Elements	MoO_2.3_/H-diamond *x* = 0.7	MoO_2.6_/H-diamond *x* = 0.4	MoO_2.9_/H-diamond *x* = 0.1
C	–0.012	–0.004	–0.003
H	0.037	0.023	0.023
Mo	–0.021	0.005	–0.013
O	–0.012	–0.025	–0.016

To further visualize the nature of charge transfer due to deposition, the charge density difference, Δρ, is computed using MoO_2.9_ as an example. In this work, charge density difference, Δρ, is defined as
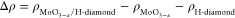
1where ρ_MoO_3–*x*_/H-diamond_ is the charge density of oxide-deposited hydrogenated diamond surface, while ρ_MoO_3–*x*__ and ρ_H-diamond_ represent the charge density of oxides and hydrogenated diamond, respectively, before deposition. In Figure 3, we observe that electrons are accumulated in oxide, while holes are accumulated in the hydrogenated diamond, especially around the surface region. We can thus safely conclude that due to deposition, electrons are extracted from diamond surface to the oxide, leading to a positively charged hydrogenated diamond and hole accumulation as expected in the surface transfer doping model.^9^ The iso-surface of the hole density in Figure 3b observed at the interface shows the holes accumulated in H-diamond. The iso-surface also signifies the spatially extended nature of doped holes in H-diamond, which is consistent with excellent transport properties of MoO_3–*x*_/H-diamond interfaces.^32^ The iso-surface is primarily located around the hydrogen atoms, which is consistent with the Bader charge analysis.

**Figure 3:**
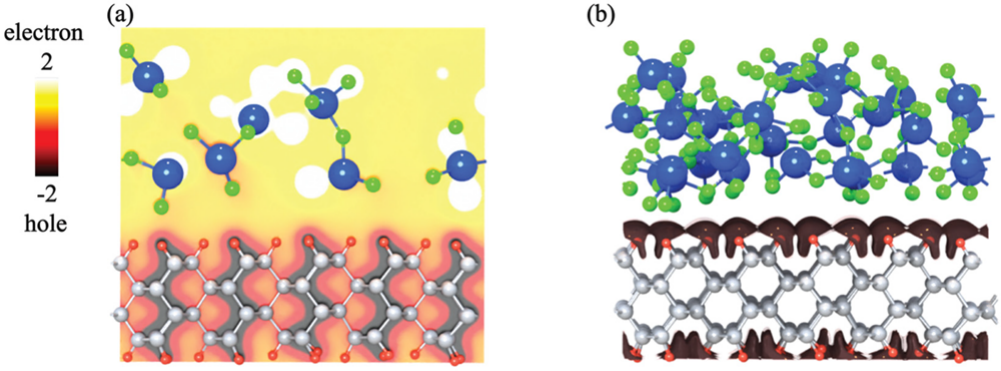
Charge density profiles for MoO_2.9_ deposited hydrogenated diamond. (a) Side view of the charge density difference of MoO_2.9_ deposited hydrogenated diamond. Yellow and brown regions represent electron and hole accumulation (electron depletion), respectively. (b) Iso-surface of charge density at the interface with iso-level of −0.08 signifies the hole distribution, shown in dark brown. The color scheme for atoms is the same as that in Figure 1.

We also compute element-projected electronic densities-of-states (PDOS) to examine the surface transfer doping process. Figure 4 shows the PDOS for hydrogenated diamonds before and after deposition of MoO_3–*x*_. We find the Fermi level shifts across the original valence band upon deposition, which ultimately depopulates the C–H σ bond at the surface. This suggests the p-type doping effect of oxides and metallic characteristic of doped diamond after deposition, which is consistent with theoretical and experimental reports.^9,33^ The energy range of holes in hydrogenated diamond is demonstrated by the dash-dotted and dashed lines, which show the Fermi energy and valence-band maximum (VBM) energy, respectively. The energy difference between the VBM and the Fermi level represents the energy range of doped holes and is a decreasing function of the vacancy level, *x*. This illustrates that the oxygen vacancy limits the hole-doping capability of MoO_3–*x*_.

**Figure 4:**
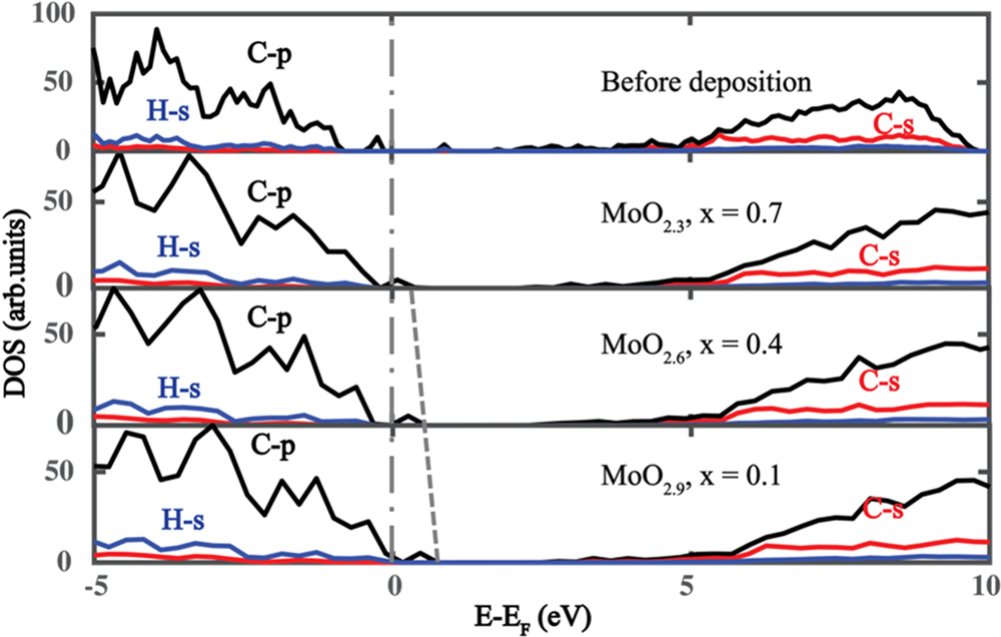
Electronic density-of-states for pristine hydrogenated diamond and MoO_3–*x*_ deposited hydrogenated diamond systems with *x* values being 0.7, 0.4, and 0.1. *E*_F_ represents the Fermi level. Gray dashed and dashed-dotted lines indicate the valence-band top and Fermi energies, respectively.

To further investigate the electronic structure, we show the entire electronic density-of-states for MoO_2.9_ deposited hydrogenated diamond alongside the density-of-states for the individual structures prior to deposition in Figure 5. When the hydrogenated diamond surface contacts the oxides, the electrons transfer from the diamond valence band to the acceptor’s conduction band minimum; therefore, the upward band bending occurs, and the surface conductivity is initiated.

**Figure 5:**
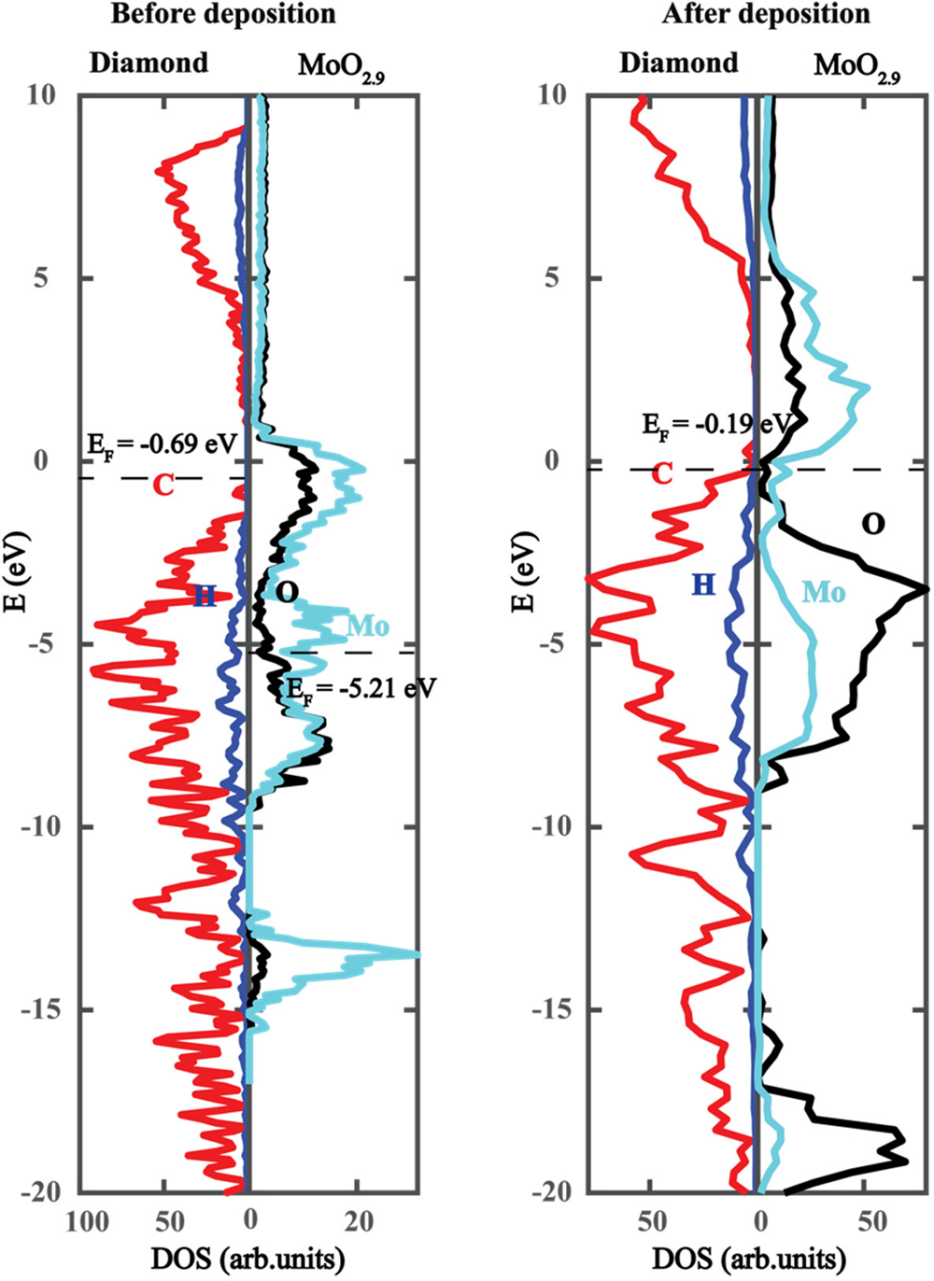
(a) Electronic density-of-states for pristine hydrogenated diamond and molten MoO_2.9_. (b) Density-of-states for MoO_2.9_ deposited hydrogenated diamond.

In this work, we performed reactive molecular dynamics simulations and density functional theory simulations to study the deposition of MoO_3–*x*_ on a hydrogenated diamond (111) surface. The difference in Bader charges after deposition revealed the net charge transfer due to deposition. Our results demonstrate that molybdenum oxide is an effective electron-accepting material, which are consistent with experiments. As the vacancy level *x* increases, the atomic arrangement becomes more compact and the Mo–O bond gets stronger. An increase in charge transfer for higher Mo oxidation state is observed, which should result in increased electrical transport in the device. This monotonic enhancement in charge transfer as a function of oxidation state provides guidance for engineering the STD process to maximize the charge transfer.

## Methods

We simulate a slab of MoO_3–*x*_ on hydrogen-terminated diamond (H-diamond) in a simulation box of dimension 15.018 × 17.830 × 30.000 Å^3^ in the *x*, *y*,and *z* directions. Periodic boundary conditions are applied in all directions where a large vacuum layer is inserted in the *z* direction to prevent periodic images from interacting. The number of oxygen atoms are 74, 82, and 93 corresponding to O/Mo ratios of 2.3, 2.6, and 2.9, respectively. The numbers of carbon, hydrogen, and molybdenum atoms are the same for different systems: 240 carbon atoms, 120 hydrogen atoms, and 32 molybdenum atoms. A three-step simulation workflow is used to optimize interfacial structure and compute electronic structure. First, we use RMD simulations to generate thermalized interfacial structure. Second, we use quantum molecular dynamics (QMD) simulation based on DFT to further optimize the RMD-created structure and then thermalize the interfacial structure. Finally, we use DFT to compute electronic structures on this equilibrated optimized structure.

### RMD to Generate Fully Thermalized Interfacial Structure

MoO_3–*x*_ is gradually heated to a temperature of 3,300 K and then allowed to melt at 3,300 K using the RXMD software.^34^ We place two “momentum mirrors” when melting the oxides. One mirror is placed 1 Å above the H-diamond surface to avoid chemical bond formation at the interface, while the other is placed on top of the oxides so that the oxide atoms will not fly away. Since the purpose of this step is to melt and fully thermalize the oxides, the carbon atoms and hydrogen atoms are fixed during this process. Subsequently, the resulting amorphous MoO_3–*x*_ is deposited on the hydrogen-terminated diamond (111) surface and the structure is relaxed at 10 K.

### DFT to Further Optimize Thermalized Interfacial Structure

QMD simulation based on DFT is performed using the VASP software^35,36^ to optimize the RMD thermalized interfacial structure. The bottom 3 layers of atoms in the H-diamond are kept fixed. We use the projector-augmented wave (PAW) method^37^ (with pseudopotentials for Mo, O, C, and H provided in VASP) and PBE generalized gradient approximation (GGA) functional,^38^ with 450 eV plane wave cutoff. The Brillouin zone was sampled over a 1 × 1 × 1 Monkhorst–Pack k-point mesh.^39^

### DFT to Compute Electronic Structures

We perform DFT calculations to compute the electronic structure of the interface to investigate the electronic density-of-states and charge transfer. For electronic structure calculations, a fine 3 × 3 × 1 Monkhorst–Pack k-point mesh^39^ is used.
